# Supplementary material for: Stronger influence of anthropogenic disturbance than climate change on century-scale compositional changes in northern forests
Source: Nat Commun. 2019 Mar 20;10:1265. doi: 10.1038/s41467-019-09265-z (PMC6426862; doi:10.1038/s41467-019-09265-z)
Supplement: Supplementary file 1 — Supplementary Information [file 41467_2019_9265_MOESM1_ESM.pdf]

Supplementary Information for

Stronger influence of anthropogenic disturbance than climate change on century-scale compositional changes in northern forests

Victor Danneyrolles, Sébastien Dupuis, Gabriel Fortin, Marie Leroyer, André de Römer, Raphaële Terrail, Mark Vellend, Yan Bouchere, Jason Laflamme, Yves Bergeron and Dominique Arseneault

Supplementary Methods

Supplementary Methods 1

Community temperature indices (ΔCTI) are based on the assignment of a temperature index to each species (STI), for which we used the median annual temperature across the geographic range of a given species, calculated from overlaid maps of interpolated mean annual surface temperature (MAT[3]) and continental-scale species distributions[4]. In order to assess the robustness of our results to the decision to use the median temperature, we tested four alternative methods to calculate STI. First, as alternate estimates of central tendency, we calculated a given species' STI as the mean MAT value and the mean MAT value after excluding outliers (i.e. values outside the species' central 95% quantile; Supplementary Table 4). Second, we calculated STI as the 10th or 90th percentile of the distribution of MAT values across the geographic range of a given species (Supplementary Table 4), thus focusing on more extreme temperatures related to tolerance of cold (10th percentile) or heat (90th percentile).

Raw values of STI obtained with all five methods (median, mean, mean 95%, 10th and 90th percentile) were highly correlated ($r>0.8$; Supplementary Table 5), indicating similar species rankings regardless of method. Overall changes in the community temperature index (ΔCTI) were almost identical using the median, mean, or mean-minus-outliers methods, and only slightly different using the 10th or 90th percentiles (Supplementary Figure 7). Using the 10th percentile as STI resulted in a slight decrease in CTI while the 90th percentile resulted in a slight increase (Supplementary Figure 7). However, ΔCTI obtained with all four methods were always of weaker magnitude than changes in disturbance-related community indices (ΔCSTI and ΔCDI; Supplementary Figure 7). Moreover, for all methods, results of mixed models showed that ΔCTI was always correlated negatively with ΔTemperature (opposite to the prediction) and such effects were mostly non-significant (Supplementary Table 6). In sum, the conclusion of disturbance effects being of far greater magnitude than climate effects is robust to these methodological decisions.

Supplementary Methods 2

Because surveyors of the 19th century did not systematically distinguish all congeneric species, 10 species (see Supplementary Tables 1 and 2) had to be combined at the genus-level (i.e., *Acer* spp., *Picea* spp., *Pinus* spp., and *Populus* spp.). Thus, species-level indices were averaged before the calculation of community-level indices. To test the sensitivity of our results to these aggregations, we built eight alternative scenarios in which index values for multi-species taxa were based on a single species in the genus rather than all species. In each scenario, we retained the species index values that seemed most likely to favour either increases or decreases over time of one of the four community indices (Supplementary Table 7). For example, in the scenario aiming to maximize the potential increase in CTI, we retained the index value of the species with the highest species temperature index for taxa that showed increases since preindustrial times (e.g., *Acer rubrum* to represent *Acer* spp., and *Populus grandidentata* to represent *Populus* spp.). Conversely, in the same scenario aiming to maximize the potential increase in CTI, we retained the index values of the species with the lowest species temperature index for taxa that showed decreases since preindustrial times (e.g., *Picea mariana* to represent *Picea* spp., and *Pinus resinosa* to represent *Pinus* spp.). Changes in community indices were then calculated for each of these 8 scenarios and compared to the results obtained with averaged multi-species values presented in the main text (the "base" scenario hereafter).

Despite the fact that our alternative scenarios are highly unrealistic in light of the modern frequencies of species (Supplementary Figure 8), relative differences among functional community indices in terms of change over time were strongly consistent across scenarios. In all eight scenarios, the magnitude of ΔCDI was always markedly higher than the magnitudes of changes in other indices (Supplementary Figure 9). The magnitude of ΔCSTI was also almost always higher than the magnitudes of changes in climate-related community indices (i.e., ΔCTI and ΔCDTI; Supplementary Figure 9). Moreover, for all eight scenarios, results of mixed models showed that effects of ΔTemperature on ΔCTI and of ΔSPEI on ΔCDTI were always opposite in direction to predictions, and such effects were mostly non-significant (Supplementary Table 8). Conversely, effects of ΔPopulation on ΔCSTI and ΔCDI were consistent and significant ($P<0.01$) in all scenarios. Our results are thus clearly robust to alternate methods of calculating taxon-level indices.

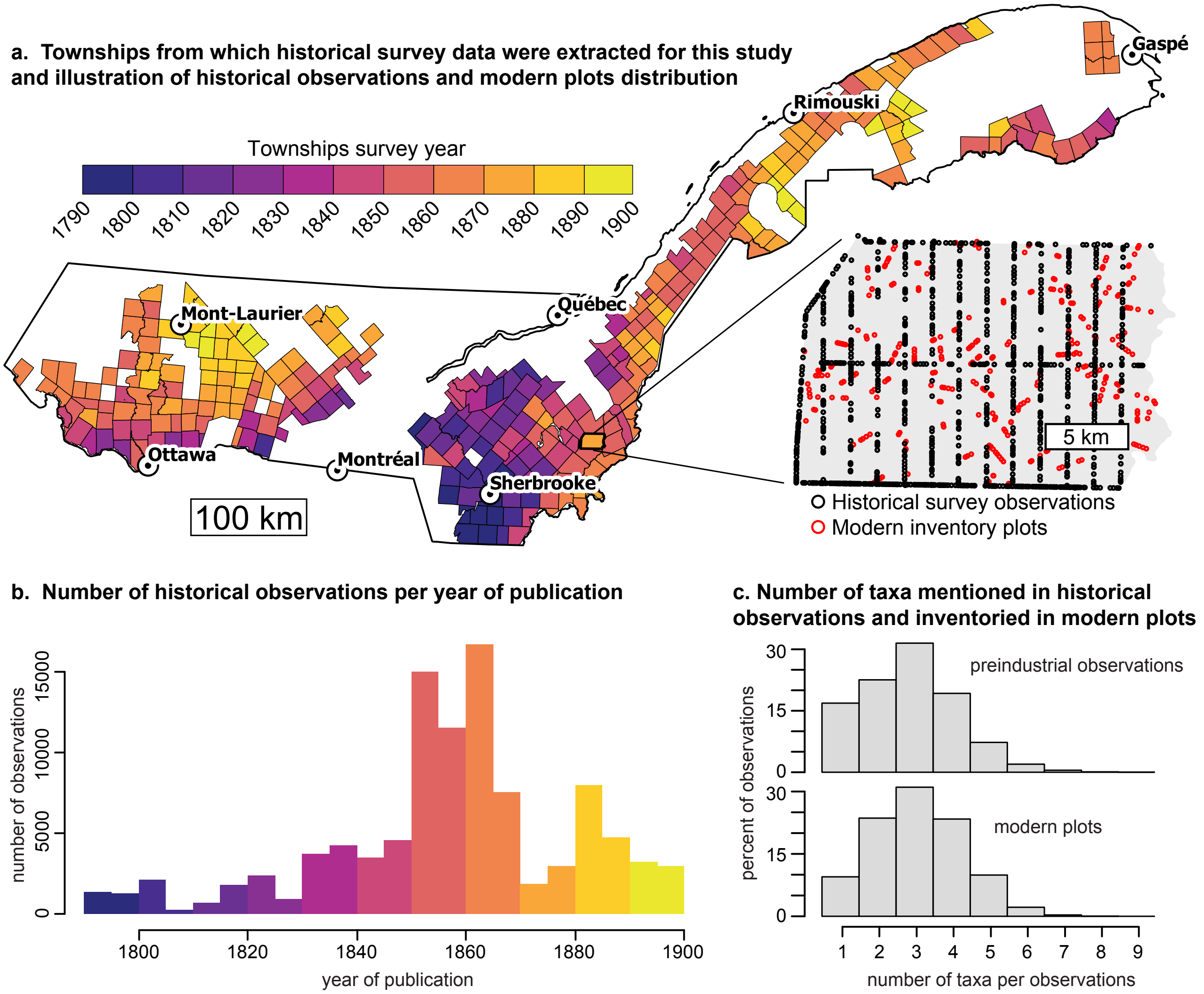

Supplementary Figure 1. Description of early land survey and modern data-sets. Map (a) shows the decades during which each township used in this study was surveyed between 1790 and 1900 and an illustration of the spatial distribution of early land survey observations and modern plot inventories within one township. Survey observations (black dots) were collected along the boundaries of the townships and along ranges lines (spaced 1.6km apart), where surveyors described the forest composition in the form of lists of taxa (e.g., “hemlock, beech and maples”). In total, 103,011 lists of taxa were precisely georeferenced using historical and modern digital cadastral maps. Modern inventory plots (red dots; 1980-2010) were distributed through stratified random sampling according to the different types of productive forest. The histogram (b) shows the number of observations per year of survey. The histograms (c) show similar distributions of numbers of taxa mentioned in historical observations (i.e., taxa lists) and in modern plots after removing taxa that represented <5% of the total basal area.

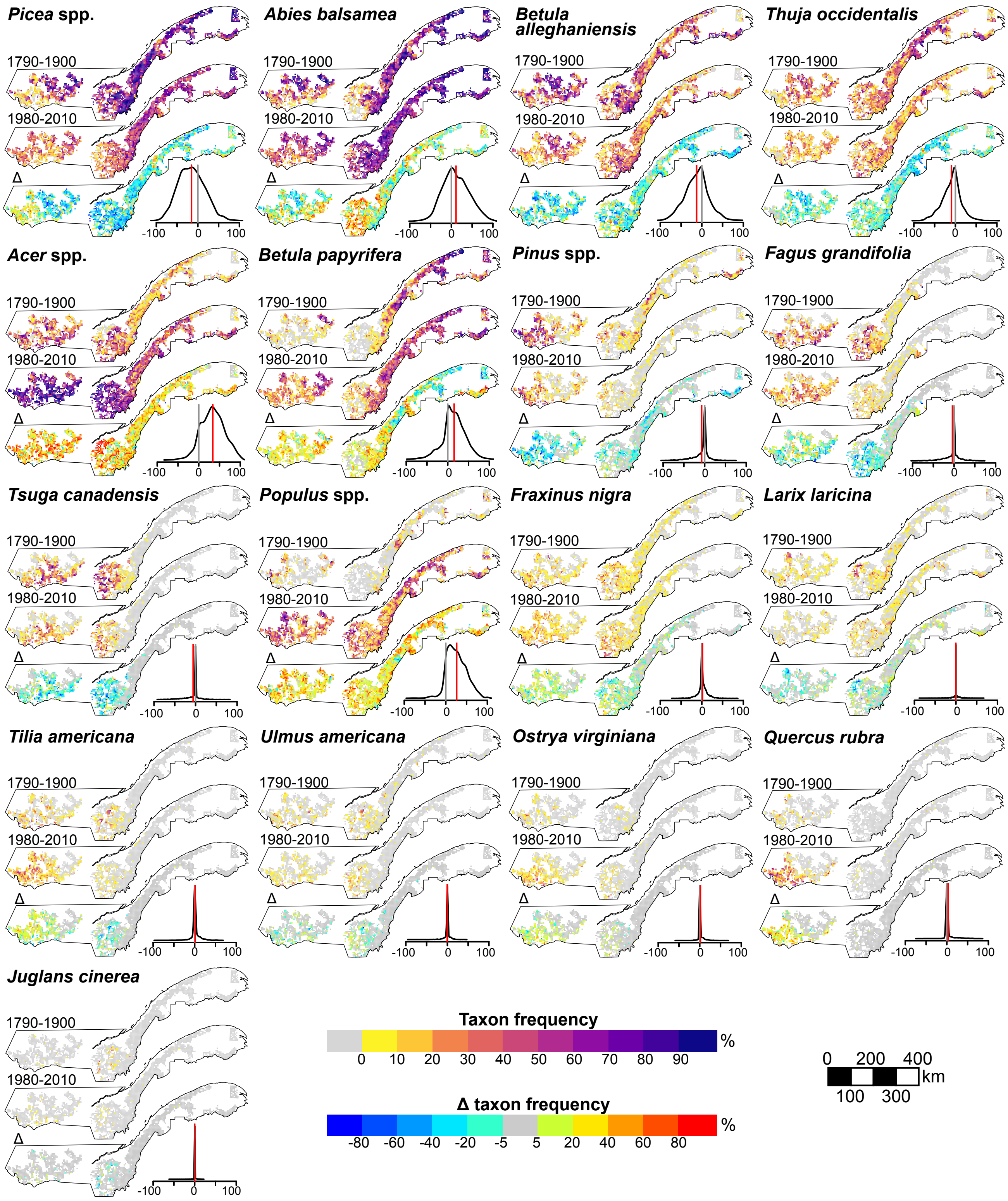

Supplementary Figure 2. Early land survey observations and modern forest inventory data aggregated to the 25km^2 grid. Maps show taxon frequencies of occurrence (%) for the 17 most abundant tree taxa in our study area, for the 1790-1900 and 1980-2010 periods, and changes between the periods (Δ). Smoothed distributions of changes are shown next to each map (vertical grey and red lines show 0 and mean values, respectively).

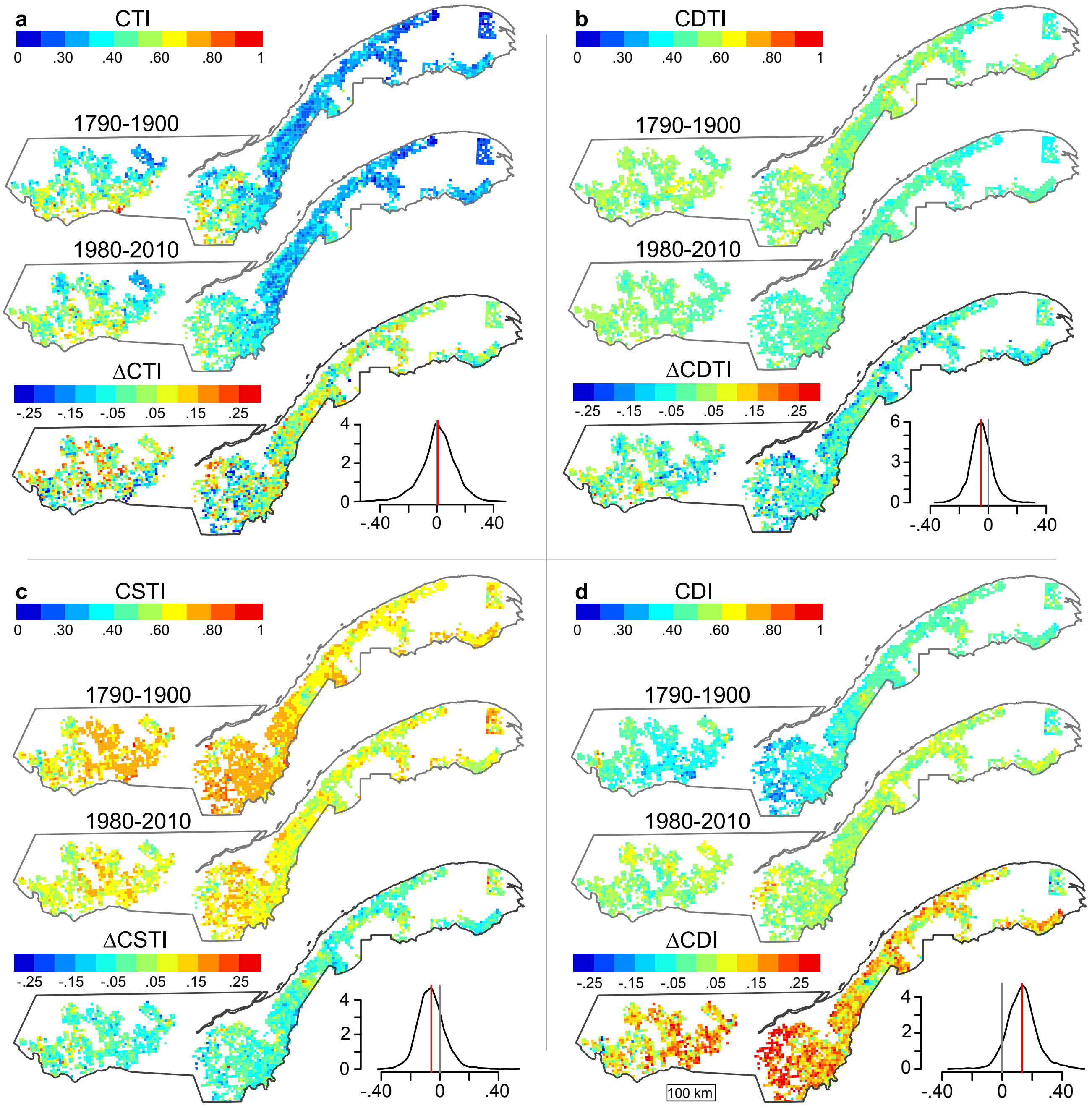

Supplementary Figure 3. Maps for the community temperature index (CTI; a), the community drought tolerance index (CDTI; b), the community shade tolerance index (CSTI; c) and the community disturbance index (CDI; d) in 1790-1900 and 1980-2010, and changes between the two time periods (the latter are also shown in Fig. 2 in the main text). Smoothed distributions of changes are shown next to each map (vertical grey and red lines show 0 and mean values, respectively).

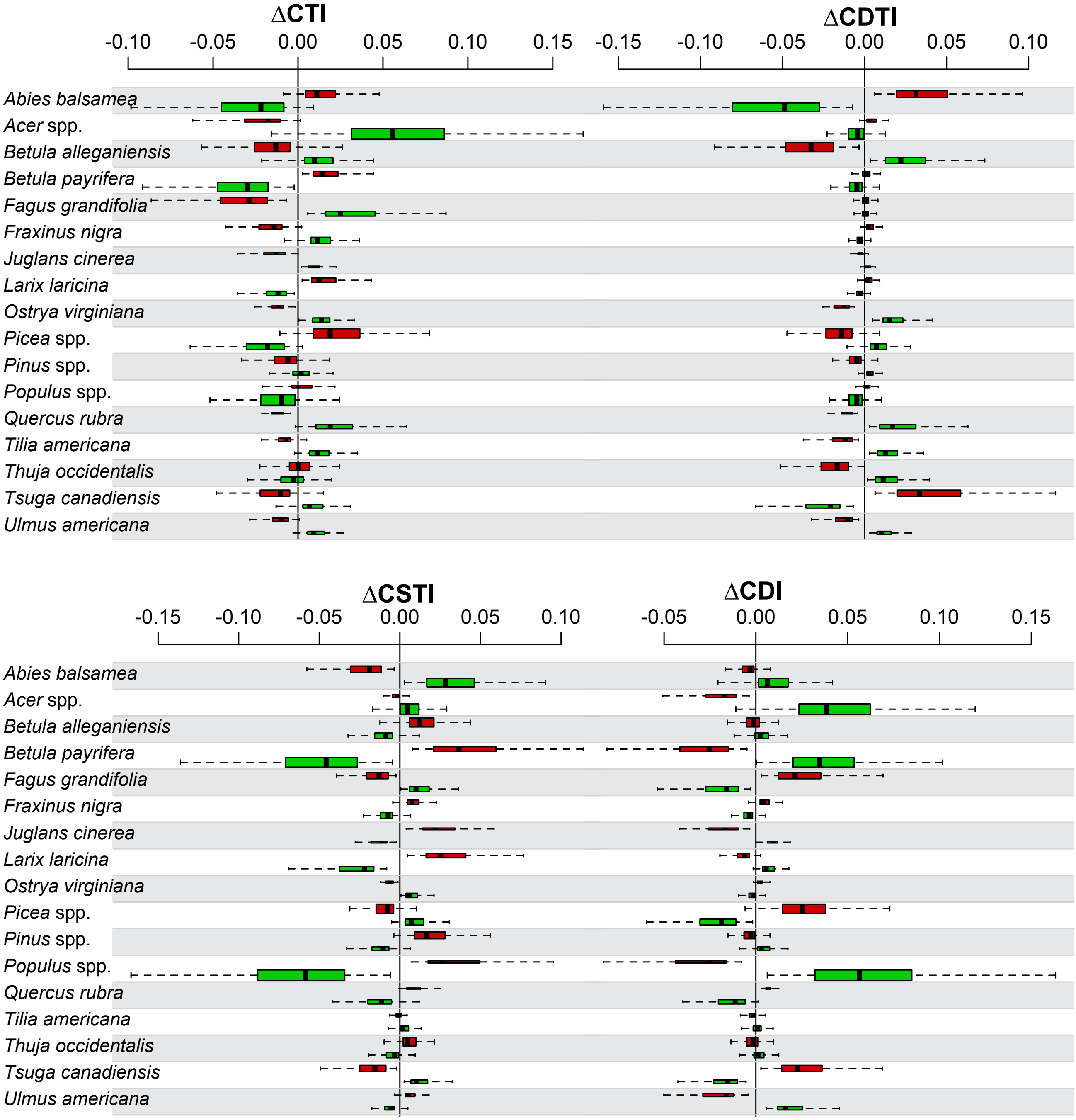

Supplementary Figure 4. Individual taxon contributions to changes in community indices (red: cells where the taxon decreased since preindustrial times (≤ -5%); green: cells where the taxon increased (≥ +5%)). The width of each box is proportional to number of cells. Following a recent study[1], for each cell, the contribution of taxon *i* to the changes in a given community index C_i was calculated as:

$$C_i = (TI_i - CI_p) \cdot (frq_{im} - frq_{ip}) / frq_m$$

where TI_i is the index for taxon *i*; CI_p is the preindustrial value of the community index, frq_{im} and frq_{ip} are, respectively, the frequencies of taxon *i* in the modern and preindustrial periods, and frq_m is the sum of taxon frequencies of the cell in modern times. It can be shown that, for one cell, the total change in CI is equal to the sum of taxa contributions. Boxes and whiskers show the 10, 25, 50, 75 and 90 percentiles of taxa contributions.

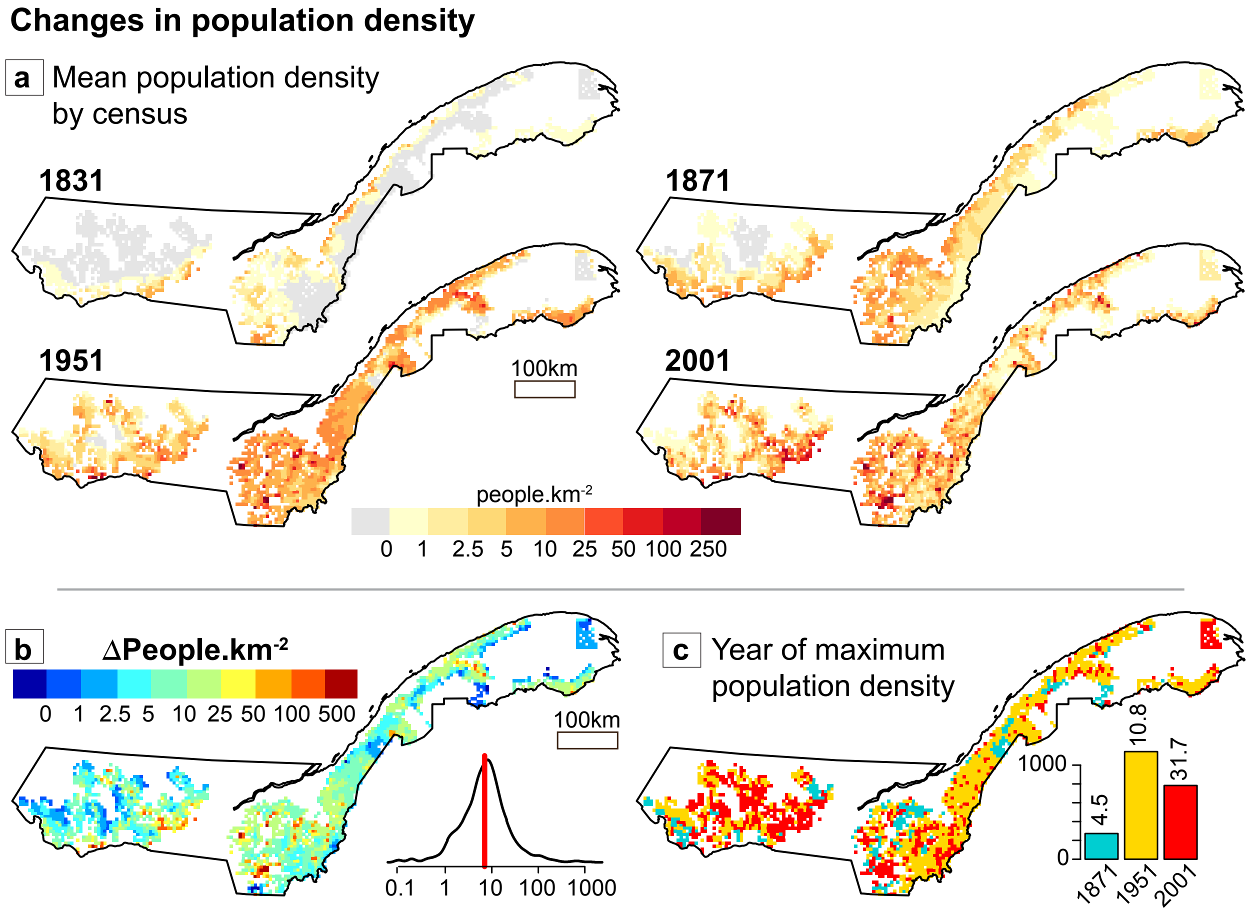

Supplementary Figure 5. Changes in human population density over time. Maps in (a) show mean population density per cell as recorded in the 1831, 1871, 1951 and 2001 censuses. Map (b) shows the difference between population density in 1831 and the maximum population density recorded in 1871, 1951 or 2001. Map (c) shows the year of maximum population density for each cell. The histogram in (c) shows the number of cells in each year class, while the numbers above the bars indicate the mean increase in population density (ΔPopulation) for each year class. Changes in population density were log-transformed prior to analysis in order to increase normality of the data. A few cells (N=10) had very small negative values for ΔPopulation (between -0.79 and -0.04 people.km^{-2}), in which case we first made ΔPopulation=0.001. All population density data were extracted from census subdivision population maps. Data from the 1831 and 1871 censuses were graciously made available by the Centre Interuniversitaire d'Études Québecoises (CIEQ; Projet GÉORIA, version 2003) and data from the 1951 and 2001 censuses are freely available on the Canada's Century Research Infrastructure (CCRI) website (https://ccri.library.ualberta.ca).

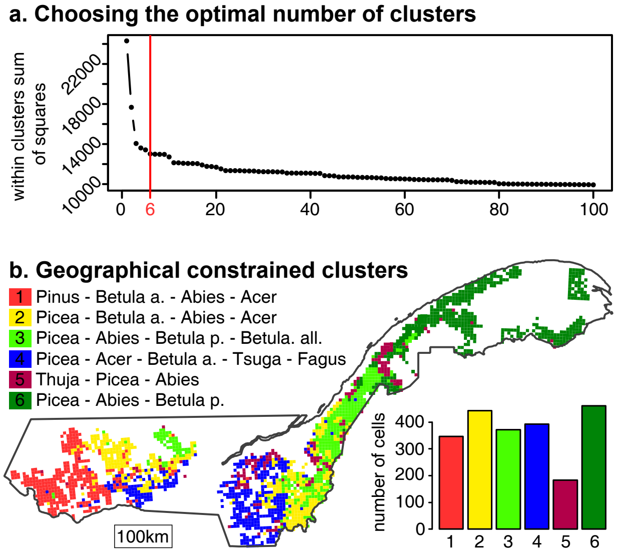

c. Clusters composition

Taxa	Clusters 1	2	3	4	5	6
Picea spp.	17.9	67.8	77.5	46.2	45.9	69.4
Abies balsamea	32.6	62.6	73.0	10.8	19.9	73.9
Betula alleghaniensis	32.4	64.1	32.4	38.1	8.1	36.8
Thuja occidentalis	20.0	32.2	27.9	18.6	45.5	35.9
Acer spp.	27.7	35.0	11.1	40.2	7.6	12.6
Betula papyrifera	5.0	5.9	36.8	1.2	4.4	49.2
Pinus spp.	41.9	7.2	10.8	4.8	8.4	6.9
Fagus grandifolia	15.3	15.6	2.4	30.8	1.8	1.1
Tsuga canadiensis	16.7	12.2	1.0	33.9	5.5	0.0
Fraxinus nigra	8.0	6.3	2.4	7.2	6.3	1.4
Populus spp.	8.5	2.2	8.0	0.4	0.6	3.9
Larix laricina	6.7	3.2	4.5	2.7	9.1	0.8
Tilia americana	6.5	2.6	0.0	6.8	0.3	0.0
Ulmus americana	4.1	1.7	0.2	4.0	0.4	0.1
Ostrya virginiana	2.6	0.7	0.0	0.3	0.0	0.0
Quercus rubra	2.8	0.1	0.0	0.3	0.0	0.0
Juglans cinerea	0.2	0.3	0.0	1.7	0.0	0.0

Supplementary Figure 6. Spatially constrained clustering of preindustrial forest composition used as a random effect in linear mixed models. We used the *hclustgeo* function from the *ClustGeo* 2.0 package[2] available in R. The function computes geographical constrained Ward-like hierarchical clustering and uses two dissimilarity matrices as input and a "mixing parameter alpha" between 0 and 1 (importance of constraint matrix). A Bray-Curtis dissimilarity matrix of the cells' preindustrial composition was used as the main input, constrained by the cells' geographical distance matrix. We first ran an unconstrained Ward-like hierarchical clustering on the Bray-Curtis dissimilarity matrix alone to choose an optimal number of 6 cluster groups, which minimize the number of groups as well as the intra-groups variation in composition (a). We finally used a mixing alpha parameter of 0.4, which maximized geographical contiguity of groups without important loss in intra-groups homogeneity. Map (b) shows the results of clustering and table (c) shows the mean frequency of each taxon (%) in the six retained groups.

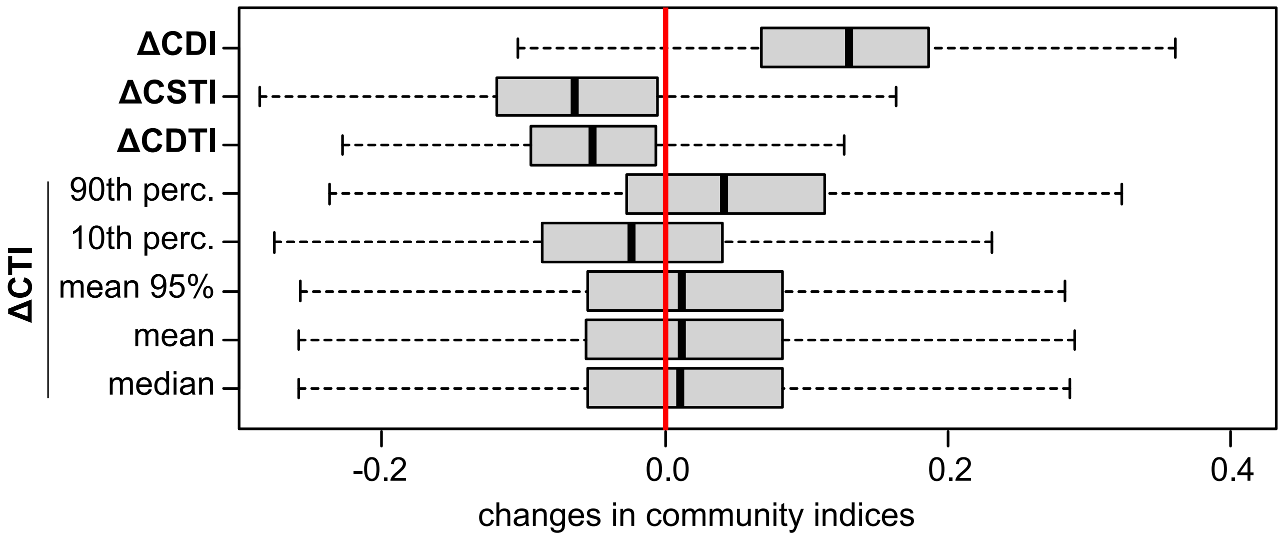

Supplementary Figure 7. Comparison of ΔCTI values obtained using different methods to compute species temperature indices. Values are also compared with values of ΔCDTI, ΔCSTI and ΔCDI present-ed in the main text. Boxes and whiskers show the 10, 25, 50, 75 and 90 percentiles of changes in community indices.

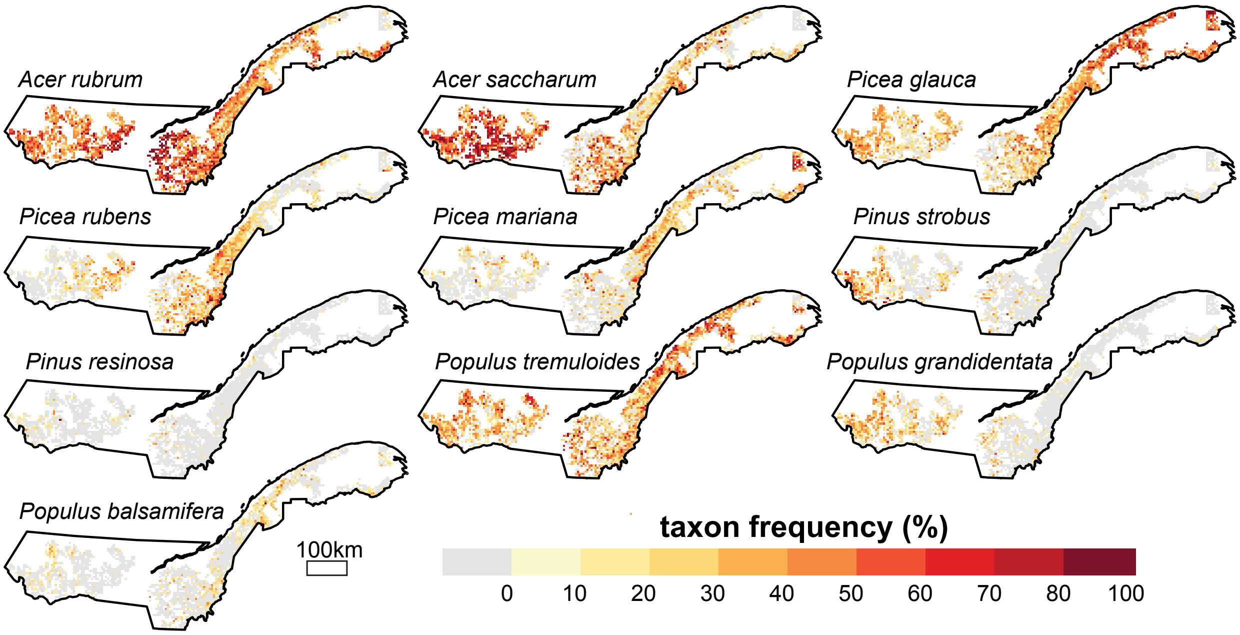

Supplementary Figure 8. Modern frequencies (1980-2010) of species grouped by genus in the analyses.

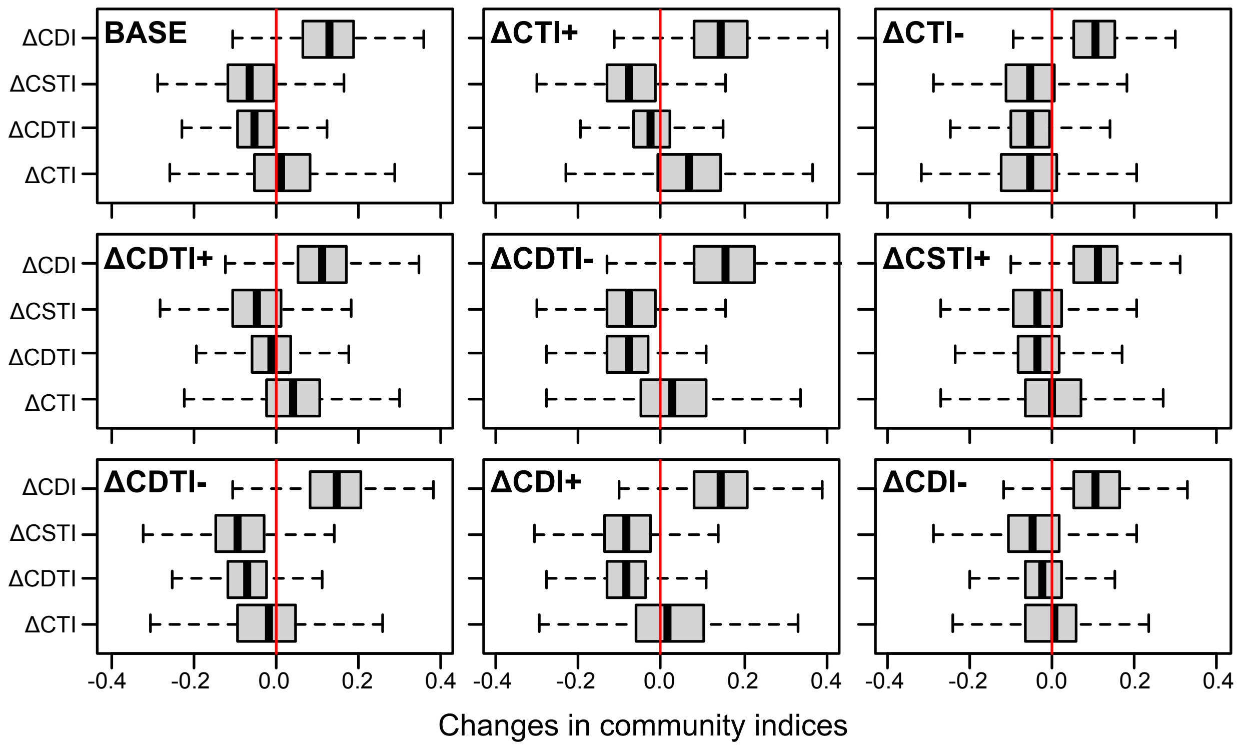

Supplementary Figure 9. Changes in community-level indices in the "base" scenario (reported in main text) plus the eight scenarios shown in Table S2.4. Boxes and whiskers show the 10, 25, 50, 75 and 90 percentiles of changes in community indices.

Supplementary Table 1. Raw values of species temperature indices (STI), species drought tolerance indices (SDTI), species shade tolerance indices (SSTI) and values of traits that were used for the calculation of species disturbance indices (SDI). To compute SDI, the 11 trait values (from SSTI to 'response to release') were first standardized to vary from 0 to 1 and then summed. For qualitative traits, values shown in parentheses were used in calculations; values of longevity and sexual maturity were inverted before the summation. (*Fruiting frequency: 1 = highly irregular at > 3-year intervals, 2 = moderately consistent to moderately irregular at 2–3-year intervals; 3 = relatively consistent and peaking at approximately a 2-year interval; 4 = good crop in most years and abundant every 1–2 years, 5 = prolific fruiting every year.)

**Species**	**STI** (°C)	**SDTI**	**SSTI**	**growth rate**	**longevity** (yr.)	**vegetative reproduction**	**sexual maturity** (yr.)	**fruiting frequency***	**seed abundance**	**seed effective dispersal** (m)	**germination substrate requirement**	**seedling vigour**	**response to release**	**SDI**
Abies balsamea	0.29	1.00	5.01	Slow (1)	150	None (1)	30	2	High (3)	40	Inter. (2)	High (3)	Strong (2)	5.09
Acer rubrum	10.22	1.84	3.25	Fast (3)	150	Slow (2)	10	4	High (3)	100	Gener. (3)	Med. (2)	Strong (2)	8.05
Acer saccharum	7.13	2.25	4.76	Slow (1)	300	Slow (2)	40	2	High (3)	100	Gener. (3)	High (3)	Strong (2)	5.40
Betula alleghaniensis	5.42	3.00	3.17	Fast (3)	300	Slow (2)	40	2	Med. (2)	100	Spec. (1)	Med. (2)	Strong (2)	4.79
Betula papyrifera	-1.21	2.02	1.54	Fast (3)	150	Slow (2)	20	3	High (3)	200	Spec. (1)	Med. (2)	Strong (2)	7.23
Fagus grandifolia	10.71	2.18	4.75	Slow (1)	250	Slow (2)	40	1	Med. (2)	30	Inter. (2)	Low (1)	Good (1)	2.23
Fraxinus nigra	8.28	2.00	2.96	Slow (1)	150	Slow (2)	30	1	Low (1)	30	Inter. (2)	Low (1)	Strong (2)	3.83
Juglans cinerea	9.59	2.38	1.88	Fast (3)	80	Slow (2)	20	2	Med. (2)	10	Gener. (3)	High (3)	Good (1)	6.83
Larix laricina	-1.21	2.00	0.98	Fast (3)	150	None (1)	40	1	High (3)	50	Spec. (1)	Med. (2)	Strong (2)	5.66
Ostrya virginiana	9.90	3.25	4.58	Slow (1)	100	Slow (2)	30	3	Low (1)	50	Gener. (3)	Med. (2)	Good (1)	4.20
Picea glauca	-2.21	2.88	4.15	Slow (1)	200	None (1)	30	1	Low (1)	100	Gener. (3)	Low (1)	Good (1)	2.45
Picea mariana	-2.52	2.00	4.08	Slow (1)	200	None (1)	30	1	Med. (2)	80	Inter. (2)	Med. (2)	Good (1)	2.93
Picea rubens	5.18	2.50	4.39	Mod. (2)	300	None (1)	30	1	Med. (2)	100	Gener. (3)	Med. (2)	Good (1)	3.44
Pinus resinosa	4.13	2.29	1.89	Fast (3)	200	None (1)	40	1	Med. (2)	10	Spec. (1)	High (3)	Strong (2)	5.13
Pinus strobus	5.19	2.38	3.21	Fast (3)	300	None (1)	20	1	Med. (2)	100	Inter. (2)	Med. (2)	Strong (2)	4.93
Populus balsamifera	-1.20	1.77	1.27	Fast (3)	130	Fast (3)	20	5	High (3)	500	Inter. (2)	Med. (2)	Strong (2)	9.50
Populus grandidentata	5.82	2.50	1.21	Fast (3)	150	Slow (1)	20	2	High (3)	500	Spec. (2)	Med. (2)	Strong (2)	7.67
Populus tremuloides	-0.22	1.77	1.21	Fast (3)	150	Fast (3)	20	1	High (3)	500	Inter. (2)	Low (1)	Strong (2)	7.92
Quercus rubra	9.16	2.88	2.75	Mod. (2)	250	Slow (2)	30	1	Med. (2)	30	Spec. (1)	Med. (2)	Good (1)	3.43
Thuja occidentalis	2.64	2.71	3.45	Slow (1)	300	Slow (2)	30	2	Med. (2)	45	Gener. (3)	Med. (2)	Strong (2)	4.81
Tilia americana	7.95	2.88	3.98	Mod. (2)	150	Slow (2)	20	3	High (3)	10	Spec. (1)	Med. (2)	Good (1)	4.74
Tsuga canadensis	6.73	1.00	4.83	Slow (1)	300	None (1)	60	3	High (3)	30	Spec. (1)	Med. (2)	Good (1)	2.09
Ulmus americana	8.51	2.92	3.14	Fast (3)	200	Slow (2)	30	5	High (3)	100	Gener. (3)	Med. (2)	Strong (2)	7.70

Supplementary Table 2. Standardized values of taxon temperature indices (TTI), taxon drought tolerance indices (TDTI), taxon shade tolerance indices (TSTI) and taxon disturbance indices (TDI) that were used to compute functional community indices, and changes in taxon frequencies between 1790-1900 and 1980-2010 (values show mean frequency ± standard deviation across 25km^2 cells, in %). To compute values of taxon-level indices, raw values of species indices (Supplementary Table 1) were first averaged for multi-species taxa (i.e.: *Acer* spp., *Picea* spp., *Pinus* spp. and *Populus* spp.) and then all indices were standardized from 0 to 1.

**Taxa**	**Taxa index**				**Changes in taxon frequency (%)**		
	**TTI**	**TDTI**	**TSTI**	**TDI**	**1790-1900**	**1980-2010**	**Δ**
Abies balsamea	0.13	0.00	1.00	0.48	49.2 ± 31.9	60.4 ± 23.8	11.2 ± 31.3
Acer spp.	0.83	0.46	0.75	0.74	23.7 ± 22.7	57.5 ± 27.3	33.7 ± 27.7
Betula alleghaniensis	0.56	0.89	0.54	0.43	38.7 ± 27.2	26.1 ± 21.3	-12.6 ± 27.4
Betula papyrifera	0.00	0.45	0.14	0.82	19.1 ± 26.9	34.4 ± 23.3	15.3 ± 26.4
Fagus grandifolia	1.00	0.52	0.94	0.02	11.8 ± 18.6	7.9 ± 13.4	-4.0 ± 18.3
Fraxinus nigra	0.80	0.44	0.49	0.28	5.1 ± 8.9	6.7 ± 9.7	1.6 ± 12
Juglan cinerea	0.91	0.61	0.22	0.75	0.4 ± 3.1	0.3 ± 1.6	-0.1 ± 3.4
Larix laricina	0.00	0.44	0.00	0.57	3.9 ± 9.2	3.2 ± 7.0	-0.6 ± 10.6
Ostrya virginiana	0.93	1.00	0.89	0.34	0.6 ± 3.0	2.3 ± 6.0	1.7 ± 6.4
Picea spp.	0.11	0.65	0.80	0.14	56.2 ± 29.8	40.9 ± 22.8	-15.3 ± 30.9
Pinus spp.	0.49	0.59	0.39	0.47	12.9 ± 20.5	5.5 ± 11.7	-7.3 ± 17.9
Populus spp.	0.22	0.45	0.06	1.00	4.1 ± 11.1	30.2 ± 22.1	26.2 ± 23.2
Quercus rubra	0.87	0.84	0.44	0.21	0.5 ± 3.3	3.7 ± 11.0	3.2 ± 10.7
Thuja occidentalis	0.32	0.76	0.61	0.43	29.0 ± 22.0	19.5 ± 18.2	-9.5 ± 22.9
Tilia americana	0.77	0.84	0.74	0.42	2.8 ± 8.2	4.0 ± 9.0	1.3 ± 10.6
Tsuga canadiensis	0.67	0.00	0.96	0.00	11.8 ± 20.0	6.0 ± 12.7	-5.8 ± 16.6
Ulmus americana	0.82	0.85	0.54	0.89	1.8 ± 6.0	1.6 ± 4.8	-0.3 ± 6.8

Supplementary Table 3. Correlations between taxon-level indices used to compute community functional composition indices. Bold values in the upper right-side show Pearson coefficients while italic values on the lower left side show Spearman's rank coefficients.

	**TTI**	**TDTI**	**TSTI**	**TDI**
**TTI**	-	**0.37**	**0.37**	**-0.26**
**TDTI**	*0.42*	-	**-0.12**	**0.08**
**TSTI**	*0.29*	*0.03*	-	**-0.64**
**TDI**	*-0.29*	*-0.07*	*-0.59*	-

Supplementary Table 4. Raw species temperature index values (°C) calculated as the median, mean, mean of values inside the central 95% quantiles (mean 95%), 10th percentile and 90th percentile of the distribution of annual temperatures across the species geographic range.

	Median	Mean	Mean 95%	10th percentile	90th percentile
Abies balsamea	0.29	0.70	0.68	-2.85	5.17
Acer rubrum	10.22	10.58	10.52	3.20	18.26
Acer saccharum	7.13	7.52	7.52	2.70	12.67
Betula alleghaniensis	5.42	5.70	5.64	2.20	9.53
Betula papyrifera	-1.21	-0.93	-0.98	-5.81	4.47
Fagus grandifolia	10.71	11.03	11.03	4.74	17.83
Fraxinus nigra	8.28	8.76	8.71	1.62	16.54
Juglans cinerea	9.59	9.54	9.54	6.01	13.07
Larix laricina	-1.21	-0.84	-0.92	-5.56	4.85
Ostrya virginiana	9.90	10.35	10.22	4.08	17.35
Picea glauca	-2.21	-1.98	-2.01	-6.54	2.75
Picea mariana	-2.52	-2.32	-2.36	-7.31	2.94
Picea rubens	5.18	5.19	5.13	3.04	7.12
Pinus resinosa	4.13	4.11	4.10	1.40	6.90
Pinus strobus	5.19	5.63	5.55	1.67	10.45
Populus balsamea	-1.20	-1.01	-1.06	-5.47	3.81
Populus grandidentata	5.82	5.87	5.84	1.64	10.07
Populus tremuloides	-0.22	0.06	0.01	-5.58	6.60
Quercus rubra	9.16	9.19	9.17	3.55	15.01
Thuja occidentalis	2.64	3.02	2.96	-0.18	6.85
Tilia americana	7.95	8.02	8.01	3.71	12.41
Tsuga canadiensis	6.73	7.36	7.31	3.96	11.80
Ulmus americana	8.51	9.15	9.09	1.87	17.56

Supplementary Table 5. Correlations between alternative methods to compute taxon-level temperature indices. Values in the upper right-side show Pearson coefficients (r) while italic values on the lower left side show Spearman's rank coefficients (ρ).

	Median	Mean	Mean 95%	10th percentile	90th percentile
Median	.	0.999	0.999	0.950	0.955
Mean	*0.997*	.	1.000	0.945	0.963
Mean 95%	*0.997*	*1.000*	.	0.946	0.962
10th percentile	*0.911*	*0.907*	*0.907*	.	0.824
90th percentile	*0.936*	*0.944*	*0.944*	*0.789*	.

Supplementary Table 6. Slopes and P-values obtained through mixed models testing the effects of Δ Temperature on ΔCTI, with the latter calculated using alternative methods for computing STIs (see Methods).

	Single predictor models		Models corrected with ΔCDI and ΔCSTI	
STI Method	slope	*P*	slope	*P*
Median	-0.0080	0.3716	-0.0204	0.0107
Mean	-0.0078	0.3854	-0.0205	0.0103
Mean 95%	-0.0077	0.3888	-0.0205	0.0102
10th percentile	-0.0040	0.5994	-0.0122	0.0683
90th percentile	-0.0094	0.3172	-0.0250	0.0031

Supplementary Table 7. Species retained for each of the four multi-species taxa in eight different scenarios aimed at maximizing change in a given direction (+ or -) for a given community-level index (left column). The first three rows show mean multi-species taxon frequencies (in $25 km^2$ cells) in preindustrial and modern times, and changes in mean taxon frequency.

	Acer spp.	*Picea* spp.	*Pinus* spp.	*Populus* spp.
Preindustrial	23.74	56.24	12.89	4.06
Modern	57.49	40.93	5.54	30.23
Δ	33.75	-15.31	-7.34	26.17
ΔCTI+	*A. rubrum*	*P. mariana*	*P. resinosa*	*P. grandidentata*
ΔCTI-	*A. saccharum*	*P. rubens*	*P. strobus*	*P. balsamifera*
ΔCDTI+	*A. saccharum*	*P. mariana*	*P. strobus*	*P. grandidentata*
ΔCDTI-	*A. rubrum*	*P. glauca*	*P. resinosa*	*P. tremuloides*
ΔCSTI+	*A. saccharum*	*P. mariana*	*P. resinosa*	*P. balsamifera*
ΔCSTI-	*A. rubrum*	*P. rubens*	*P. strobus*	*P. tremuloides*
ΔCDI+	*A. rubrum*	*P. glauca*	*P. strobus*	*P. balsamifera*
ΔCDI-	*A. saccharum*	*P. rubens*	*P. resinosa*	*P. grandidentata*

Supplementary Table 8. Slopes and P-values obtained through mixed models testing relationships between community indices and potential predictors (effects of ΔTemperature on ΔCTI, of ΔSPEI on ΔCDTI, and of population density on both ΔCSTI and ΔCDI) for the base scenario (see main text) and the eight scenarios shown in Table S2.4.

	ΔCTI		ΔCDTI		ΔCSTI		ΔCDI	
	slope	*P*	slope	*P*	slope	*P*	slope	*P*
BASE	-0.008	0.3716	0.007	0.0315	-0.007	0.0005	0.007	0.0008
ΔCTI+	0.000	0.9787	0.003	0.4271	-0.008	0.0002	0.007	0.0054
ΔCTI-	-0.017	0.0230	0.008	0.0196	-0.007	0.0017	0.007	0.0000
ΔCDTI+	-0.003	0.7035	0.002	0.6102	-0.007	0.0013	0.007	0.0002
ΔCDTI-	-0.005	0.6241	0.011	0.0026	-0.008	0.0003	0.007	0.0088
ΔCSTI+	-0.009	0.3024	0.005	0.1353	-0.008	0.0006	0.007	0.0001
ΔCSTI-	-0.012	0.1594	0.009	0.0082	-0.007	0.0012	0.007	0.0021
ΔCDI+	-0.005	0.6127	0.011	0.0026	-0.007	0.0009	0.007	0.0034
ΔCDI-	-0.012	0.0858	0.004	0.1795	-0.008	0.0007	0.008	0.0000

Supplementary References

1. Zhang, T., Niinemets, Ü., Sheffield, J. & Lichstein, J. W. Shifts in tree functional composition amplify the response of forest biomass to climate. Nature (2018). doi:10.1038/nature26152
2. Chavent, M., Kuentz-Simonet, V., Labenne, A. & Saracco, J. ClustGeo: an R package for hierarchical clustering with spatial constraints. Comput. Stat. 33, 1799–1822 (2018).
3. Hijmans, R. J., Cameron, S. E., Parra, J. L., Jones, P. G. & Jarvis, A. Very high resolution interpolated climate surfaces for global land areas. Int. J. Climatol. 25, 1965–1978 (2005).
4. Little, E. L. & Viereck, L. A. Atlas of United States trees. 5, (US Dept. of Agriculture, Forest Service, 1971).
